# Evidence That Pupil Size and Reactivity Are Determined More by Your Parents Than by Your Environment

**DOI:** 10.3389/fneur.2021.651755

**Published:** 2021-04-29

**Authors:** Abdus Samad Ansari, Jelle Vehof, Christopher J. Hammond, Fion D. Bremner, Katie M. Williams

**Affiliations:** ^1^Section of Academic Ophthalmology, School of Life Course Sciences, Faculty of Life Sciences & Medicine, King's College London, London, United Kingdom; ^2^Department of Ophthalmology and Epidemiology, University Medical Centre Groningen, University of Groningen, Groningen, Netherlands; ^3^Department of Neuro-Ophthalmology, National Hospital for Neurology and Neurosurgery, London, United Kingdom

**Keywords:** pupil size, heritability, genetics, pupil, twin study

## Abstract

**Purpose:** A classic twin study to evaluate the relative contributions of genetic and environmental factors to resting pupil size and reactivity.

**Methods:** Pupillometry was performed on 326 female twins (mean age 64 years) from the TwinsUK Adult Twin Registry, assessing resting pupil diameter in darkness and increasing levels of ambient light, alongside dynamic pupillary characteristics. Maximum-likelihood structural equation models estimated the proportion of trait variance attributable to genetic factors.

**Results:** Mean (SD) pupil diameter in darkness was 5.29 mm (0.81), decreasing to 3.24 mm (0.57) in bright light. Pupil light reaction (PLR) had a mean (SD) amplitude of 1.38 mm (0.27) and latency of 250.34 milliseconds (28.58). Pupil size and PLR were not associated with iris colour, intraocular pressure or refractive error, but were associated with age (diameter β = −0.02, *p* = 0.016, constriction amplitude β = −0.01, *p* < 0.001, velocity β = 0.03, *p* < 0.001, and latency β = 0.98, *p* < 0.001). In darkness the resting pupil size showed a MZ intraclass correlation coefficient of 0.85, almost double that of DZ (0.44), suggesting strong additive genetic effects, with the most parsimonious model estimating a heritability of 86% [95% confidence interval (CI) 79–90%] with 14% (95% CI 10–21%) explained by unique environmental factors. PLR amplitude, latency and constriction velocity had estimated heritabilities of 69% (95% CI 54–79%), 40% (95% CI 21–56%), and 64% (95% CI 48–75%), respectively.

**Conclusion:** Genetic effects are key determinants of resting pupil size and reactivity. Future studies to identify these genetic factors could improve our understanding of variation in pupil size and pupillary reactions in health and disease.

## Introduction

Pupil size is important not only because it controls the amount of light that is able to reach the retina but also because it affects the levels of chromatic and spherical aberration, thereby optimising visual perception (1). Pupil size is governed by the opposing actions of the iris sphincter and dilator muscles under the control of the parasympathetic and sympathetic nerves, respectively. In the healthy population there is significant variation in the resting size of the pupil and in the reflex constriction of the pupil to light. In any individual person pupil size is influenced by a number of factors including ambient light, retinal sensitivity, central cognitive processes (2, 3), alertness and emotional unrest (4, 5) with pupil measurements additionally being used by researchers as a biomarker of activity in the autonomic nervous system- both in health and in disease. Many studies have evaluated this variation in pupil size in different illumination levels, suggesting pupil size decreases in a linear manner for each luminance level (6). At a population level it has also been shown that pupils tend to be smaller with increasing age (7). This inverse correlation between age and resting pupil size has been confirmed pre-operatively in refractive surgery candidates across a range of different luminance levels (8, 9), but the effect seems to be most marked at low luminance suggesting that it is due to a progressive decline in sympathetic tone throughout adult life (7, 10).

Certain medical conditions such as diabetes and pseudoexfoliation can also decrease the effect of dilating drops on the size of the pupil (11). Different classes of glaucoma medication also influence pupil size (1). Although it was previously believed that pupil size may decrease significantly after cataract surgery, more recent literature suggests the reduction in size is temporary and returns to preoperative levels by 1 month (12).

In addition to this variability in resting pupil size, clinically there is wide variation in the dynamic pupillary light reflex (PLR) in healthy individuals. A more rigorous assessment of the PLR is now possible using pupillometric measurements using infrared video techniques. Measurements obtained include the amplitude of the light reflex, latency (13), velocity (peak or average) (14) and acceleration (15), all of which show wide variation in the healthy population. In part this may be due to the variation in resting pupil size, since a smaller pupil admits less of the stimulus under closed-loop conditions; an autonomic control system whereby a mechanism is regulated by feedback. However, even in studies where the light stimulus has been focussed to a size narrower than even the smallest pupil (i.e., “Maxwellian” optics which create open-loop conditions) and amplitude measurements are expressed as percentage change relative to the starting diameter, there is still a varied range in all PLR measurements across healthy individuals (16).

Over recent years, automated pupillometry has evolved into a robust and reliable measure of pupillary evaluation. This has removed the subjectivity of pupillary evaluation, providing data which is more accurate and clinically significant. Its use has been supported in the monitoring neurointensive care patients, for whom subtle changes can be a predictive factor of neurological deterioration (17). More recently a number of studies have highlighted its clinical relevance including in the evaluation of refractive surgery (18), sleep studies (19), monitoring the effects of certain medication on the central nervous system and even as a measure of emotional response in patients with psychiatric disorders (20). However, despite the significant clinical implication of understanding determinants of resting pupil size and reactivity to light, little is known about the role of genes. An improved understanding could enable clinicians to more reliably use pupil measurements to detect and monitor a wide range of diseases including glaucoma, other forms of optic neuropathy, and idiopathic intracranial hypertension (21). Often these diseases are bilateral and thus the fellow eye cannot be used as an internal control; distinguishing disease from health in these cases can be extremely challenging, instead pupil measurements must be compared with the normative database. However, the “normal ranges” for these pupil measurements are discouragingly wide, lowering the sensitivity for disease detection and the usefulness of pupil testing in clinical practise. The potentially high genetic determinate of pupil measurements will provide an improved belief on the stability of these results, their clinical implications and reduce the fear of environmental influence. Additionally, this improved understanding of pupil size and constriction may allow further appreciation of the mechanisms involved. This can assist in identifying individuals whose pupillary characteristics are different from those predicted by genetic risk scores thereby enabling earlier identification of abnormalities.

Twin studies provide us with the unique ability to examine the relative contribution of environmental and genetic factors to trait variation. Heritability is the quantification of the overall phenotypic variation that is attributable to genetic factors. Heritability estimates allow us to define boundaries for the ability of genetics to predict traits or disease and indirectly provides insights into the role of genes (22). Heritability calculations are performed by comparing the concordance of disease or covariance of quantitative traits in non-identical dizygotic (DZ) and identical monozygotic (MZ) twin pairs. Given that MZ twins share nearly 100% of their genetic data and DZ only half, any greater similarity within MZ twin pairs can be ascribed to this additional genetic sharing (11).

The purpose of this study was to undertake a classical twin study evaluating the relative contributions of genetic and environmental factors to resting pupil size under varying degrees of background illuminance and the PLR (the dynamic pupil response to a transient light stimulus).

## Materials and Methods

### Participants

Healthy twin volunteers were recruited from the TwinsUK registry based at St Thomas' Hospital, London (23). Twin participants attending for ocular examinations as part of ongoing studies were additionally recruited to undergo pupillometry between January 2010 and April 2011. Pupillometry was used to assess the resting pupil diameter in darkness and in increasing levels of ambient light, and also the dynamic pupillary response to a standardised transient light stimulus. Additional information was collected on patient demographics (age at examination, sex), iris colour (brown, blue or green), non-cycloplegic autorefraction (ARM-10 autorefractor, Takagi Seiko, Japan), intra-ocular pressure (non-contact tonometry using Visionix PT100), past ocular history, past medical history (including neurological disorders and diabetes) and a complete list of current medications including any topical eye drops. Twin zygosity was determined either by genotyping or through the “peas in the pod” questionnaire (PPQ). The PPQ questionnaire has previously been validated for its excellent accuracy as a proxy indicator of zygosity in the absence of genotyping information (24). Spherical equivalent was calculated in the standard manner for each eye as sphere + (cylinder/2).

Inclusion criteria included a minimum age of 18, the capacity to understand the study and consent to participate. Patients were excluded if they were a known diabetic given the well-accepted association with autonomic pupillary dysfunction. The study had appropriate local research ethics approval (EC04/015) and was conducted in accordance with the tenets of the Declaration of Helsinki.

### Pupillometry and Protocols

All pupillometry measurements were taken using the Procyon P3000 pupillometer (Procyon Instruments Ltd.). This is a binocular dynamic pupilometer that allows for customised stimulus protocols and uses video cameras running at 25 Hz with a spatial resolution of 0.03 mm. This device and similar protocols have previously been described elsewhere (25). In brief, each study participant had the resting pupil diameter measured in increasing levels of ambient light: “dark,” “low,” “medium,” and “high” (labelled as resting states 1–4, respectively); the subject was equilibrated to each ambient light level for 30 s before measuring the resting pupil size (averaged over 3 s). The background illuminance levels used were the four standard light levels provided by the manufacturer and “factory calibrated” such that the illumination at the cornea was ~0.00 Lux (“dark”), 0.04 Lux (“low”), 0.40 Lux (“medium”), and 4.00 Lux (“high”). The “standard” light stimulus used to elicit the PLR = 4.00 Lux. A series of standard white light stimuli (duration 1.0 s, interstimulus interval 5 s) was then presented alternately to right and left eyes (total of three stimulus presentations to each eye) and the pupil responses averaged to measure various outcome parameters associated with the direct and consensual pupil light reflex (amplitude, latency, and maximum constriction velocity).

### Analysis

Details of twin modelling methods have been previously described (26, 27). The methodology is based on the evaluation of the variance and covariance matrices between (MZ) and (DZ) twin pairs.

The approach utilises structural equation modelling to separate the observed phenotypic variance into additive (A) genetic effects, dominant genetic effects (D) or common environmental (C) effects, and unique environmental (E) effects, the latter also including measurement error. By subsequently dividing these individual components by the total variance you are able to determine the different standardised components of variance. To adjust for the effects of age on studied pupil traits, we performed linear regression models with pupil traits and age at visit, from which we obtained the residuals. Heritability models were then performed using these residuals. Structural equation modelling is used to estimate best-fit model that explains the variance and covariance of a trait, using the phenotype information from each twin pair and knowing their genetic relationship. Only three variables can be inferred to fit the data from two twins, so an ACDE model cannot be used; ACE or ADE models have to be used separately. An ADE model implies non-additive genetic effects, which may the case when the MZ correlation is more than twice the DZ correlation- otherwise an ACE model is chosen, implying additive genetic effects explain the greater MZ twin correlation. Univariant models were initially created using all specified parameters (ACE/ADE) and then using a stepwise approach, individual parameters were dropped, testing the deterioration in model fit accordingly. This creates three parsimonious models with increasing degrees of freedom (AE, CE, and E). Twice the difference in log likelihoods between the full and sub models follows a *x*^2^ distribution, with degrees of freedom equal to the difference in degrees of freedom between the full and subsequent models (likelihood ratio test). Individual sub models are subsequently compared with the full model with the best fitting model determined by identification of the model with the lowest Akaike information criterion (AIC). The AIC is a measure of the relative quality of statistical models for a given data set. Heritability is consequently estimated from the best-fitting model from the total contribution of genetic factors to trait variance.

Statistical analysis was performed using STATA (Stata Corp., College Station, TX). Twin modelling was completed using the OpenMx (http://openmx.psyc.virginia.edu) package in R (http://www.R-project.org), with results from the left eye reported.

## Results

Three hundred eighty-five twins were considered for our study. Participants were excluded if they were unpaired or if zygosity was unknown (*n* = 7), diabetic (*n* = 30) or if incomplete measurements were obtained for the parameters investigated (*n* = 22). In total results from 326 twins were included [70 MZ (43%) and 93 DZ (57%) twin pairs] in heritability calculations. Zygosity was determined by genotyping in 94% of the population (*n* = 305) and through PPQ in 6% (*n* = 21). Mean age at time of examination was 63.7 years [Standard Deviation (±) 7.9 (range 37–81)] and all participants were female (100%). Only one twin pair was not of European ethnicity (99% European). Self-reported iris colour was available in 232 twins, of which 144 were blue (61.2%), 50 brown (22.4%) and 38 green (16.5%). Mean spherical equivalent was −0.25 D (±2.9) in the right eye and −0.25 D (±2.8) in the left eye. Mean IOP of both eyes was 15.1 mmHg (±3.3).

Regarding medical history, 1 (0.3%) had a neurological disorder and 10 (3.1%) were pseudophakic. Thirty-two patients took regular eye drops [indications included glaucoma (*n* = 5), dry eye disease (*n* = 25) and other (*n* = 2)]. Eighteen (5.5%) patients had other forms of eye surgery including squint surgery (*n* = 5), oculoplastic procedures (*n* = 6), retinal detachment surgery (*n* = 4) and undefined laser procedures (*n* = 3). There was no significant difference in the means of any of the pupil measurements between MZ and DZ twins. Full details can be seen in Table 1.

**Table 1:** Full demographic details and left eye pupil measurements of all twin participants.

	**Total (*n* = 326)**	**MZ twin participants (*n* = 140)**	**DZ twins participants (*n* = 186)**	***p*-value**
**Age (mean, sd)**	63.7 (7.9)	64.2 (8.3)	63.3 (2.7)	0.32
Range	37–81	46–80	37–81	
**Sex**				
Female (*n*, %)	326 (100%)	140 (100%)	186 (100%)	–
**Ethnicity (*****n*****, %)**				
European	324 (99.4%)	104 (100%)	196 (98.9%)	0.22
Black	2 (0.6%)	–	2 (1.1%)	
**Iris colour (*****n*****, %)**				
Brown	57 (17.5%)	26 (18.7%)	31 (16.7%)	0.52
Blue	174 (53.5%)	70 (50.34%)	104 (55.9%)	
Green	43 (13.2%)	21 (15.1%)	22 (11.8%)	
Unknown	52 (28.8%)	23 (15.8%)	29 (15.6%)	
**Ocular history (*****n*****, %)**				
Previous eye surgery	18 (5.5%)	8 (5.8%)	10 (5.4%)	0.88
Pseudophakia	10 (3.1%)	5 (3.6%)	5 (2.7%)	0.64
Regular eye drops	32 (9.9%)	14 (10.1%)	18 (9.8%)	0.51
Left eye spherical equivalent (D) (mean, sd)	−0.25 (2.83)	−0.35 (3.15)	−0.18 (2.57)	0.59
Left eye intraocular pressure (mmHg) (mean, sd)	15.02 (3.23)	14.80 (3.20)	15.19 (3.26)	0.33
**Left eye resting pupil diameter (mean, sd)**				
Resting state 1 (mm)	5.29 (0.81)	5.35 (0.79)	5.25(0.82)	0.29
Resting state 2 (mm)	4.63 (0.85)	4.64 (0.86)	4.62 (0.84)	0.79
Resting state 3 (mm)	4.05 (0.81)	4.09(0.83)	4.03(0.80)	0.53
Resting State 4 (mm)	3.24 (0.57)	3.28(0.56)	3.23(0.57)	0.30
**Left eye pupil light reflex (mean, sd)**				
Latency (ms)	250.34 (28.58)	251.54 (32.50)	249.46 (25.39)	0.53
Constriction velocity (mm/s)	−4.08 (0.80)	−4.10 (0.84)	−4.07 (0.77)	0.73
Amplitude (mm)	1.38 (0.27)	1.39 (0.28)	1.37(0.26)	0.58

*Differences between monozygotic (MZ) and dizygotic (DZ) twin pairs were assessed using unpaired t-test and Chi-square tests, for quantitative and categorical variables, respectively. No significant differences between the two groups were identified*.

**Figure 1:**
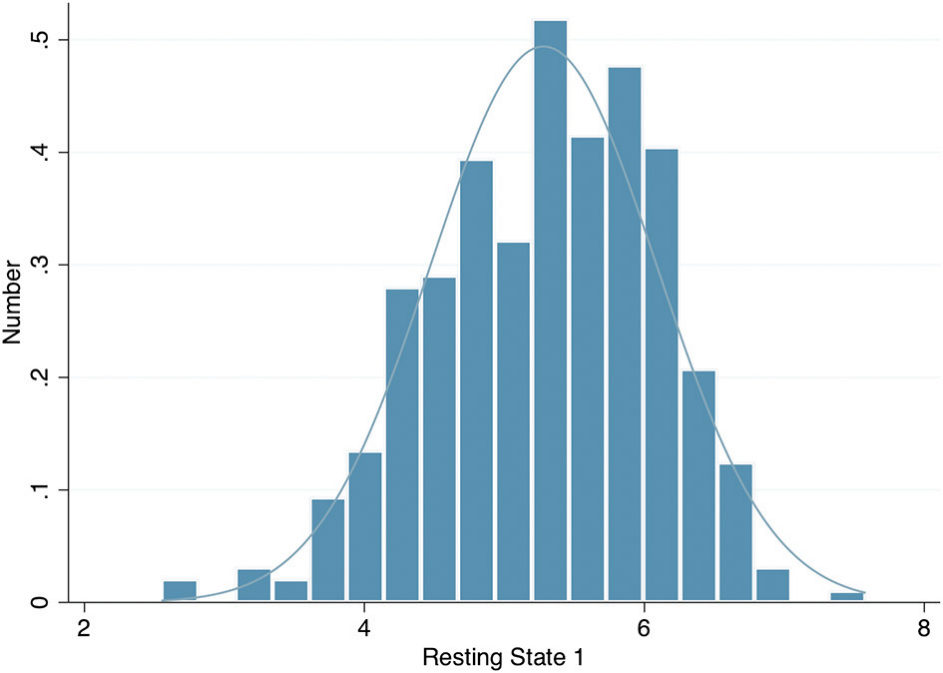
Histogram of left eye resting pupil diameter in darkness.

The left eye resting pupil size varied widely, from <3 mm to over 7 mm in darkness, with an approximately normal distribution (Figure 1); similarly, there was wide variation in the PLR measurements. However, in a linear regression model incorporating age, sex, spherical equivalent, intraocular pressure, eye colour and whilst clustering by unique family identifier, only age was significantly associated with resting pupil diameter (β = −0.02, *p* = 0.016), PLR amplitude (β = −0.01, *p* ≤ 0.001), constriction velocity (β = 0.03, *p* ≤ 0.001) and PLR amplitude (β = 0.98, *p* ≤ 0.001) (Table 2).

**Table 2:** Multivariable linear regression models examining the association of age, iris colour, intraocular pressure and spherical equivalent on pupil characteristics in the left eye, adjusted for family structure.

	**Resting state 1**		**Amplitude**		**Constriction velocity**		**Pupil latency**	
	**β (95% CI)**	***P*-value**	**β (95% CI)**	***P*-value**	**β (95% CI)**	***P*-value**	**β (95% CI)**	***P*-value**
**Age**	−0.02 (−0.03 to −0.004)	0.016	−0.01 (−0.01 to −0.004)	<0.001	0.03 (0.02 to 0.05)	<0.001	0.98 (0.45 to 1.52)	<0.001
**Iris colour**								
Brown	ref	–	ref	–	ref	–	ref	–
Blue	−0.13 (−0.45 to 0.19)	0.413	−0.05 (10.15 to 0.04)	0.258	−0.19 (−0.46 to 0.07)	0.153	5.32 (−3.19 to 14.02)	0.215
Green	0.05 (−0.32 to 0.42)	0.781	0.01 (−0.12 to 0.13)	0.925	−0.21 (−0.59 to 0.17)	0.284	−1.57 (−13.83 to 10.69)	0.801
**Intraocular pressure**	−0.01 (−0.04 to 0.03)	0.722	0.001 (−0.01 to 0.013)	0.842	−0.01 (−0.04 to 0.03)	0.775	−0.02 (−1.15 to 1.12)	0.979
**Spherical equivalent**	−0.02 (−0.06 to 0.01)	0.135	0.001 (−0.01 to 0.014)	0.829	−0.02 (−0.06 to 0.01)	0.232	−0.59 (−2.27 to 1.08)	0.485

*Complete data available for 272 individuals*.

Within our cohort individuals with blue irides had the smallest resting pupil diameter (mean 5.21 mm, ±0.77) vs. 5.52 mm (±0.80) in green irides vs. 5.35 mm (±0.90) in brown irides, *p* trend = 0.468, no difference in mean age was seen between groups (*p* trend = 0.125). The PLR constriction velocity was slowest in blue irides (mean −3.96 mm/s, ±0.81) vs. −4.29 mm/s (±0.96) in green irides vs. −4.21 mm/s (±0.69) in brown irides, but again no statistical trend difference between iris colours was observed (*p* trend = 0.669) (Table 3).

**Table 3:** Mean left eye pupil measurements for resting pupil diameter, PLR amplitude, constriction velocity (CV) and pupil latency for brown, green and blue eyes.

	**Resting state 1 (left): mean (sd, range)**	**Left eye amplitude: mean (sd, range)**	**Left eye CV max: mean (sd, range)**	**Left eye pupil latency: mean (sd, range)**
Brown (*n* = 57)	5.35 (0.90, 2.55 to 6.76)	1.42 (0.25, 0.76 to 1.99)	−4.21 (0.69, −5.39 to −2.12)	250.27 (24.31, 175 to 325)
Green (*n* = 43)	5.52 (0.80, 4.18 to 7.58)	1.45 (0.32, 0.47 to 2.21)	−4.29 (0.96, −6.61 to −1.34)	250.90 (31.6, 158 to 359)
Blue (*n* = 174)	5.21 (0.77, 3.4 to 6.8)	1.35 (0.26, 0.68 to 1.96)	−3.96 (0.81, −5.82 to −1.78)	246.26 (28.30, 188 to 328)
*P* trend	0.468	0.325	0.669	0.176

*No statistical differences between eye colours was identified*.

**Figure 2:**
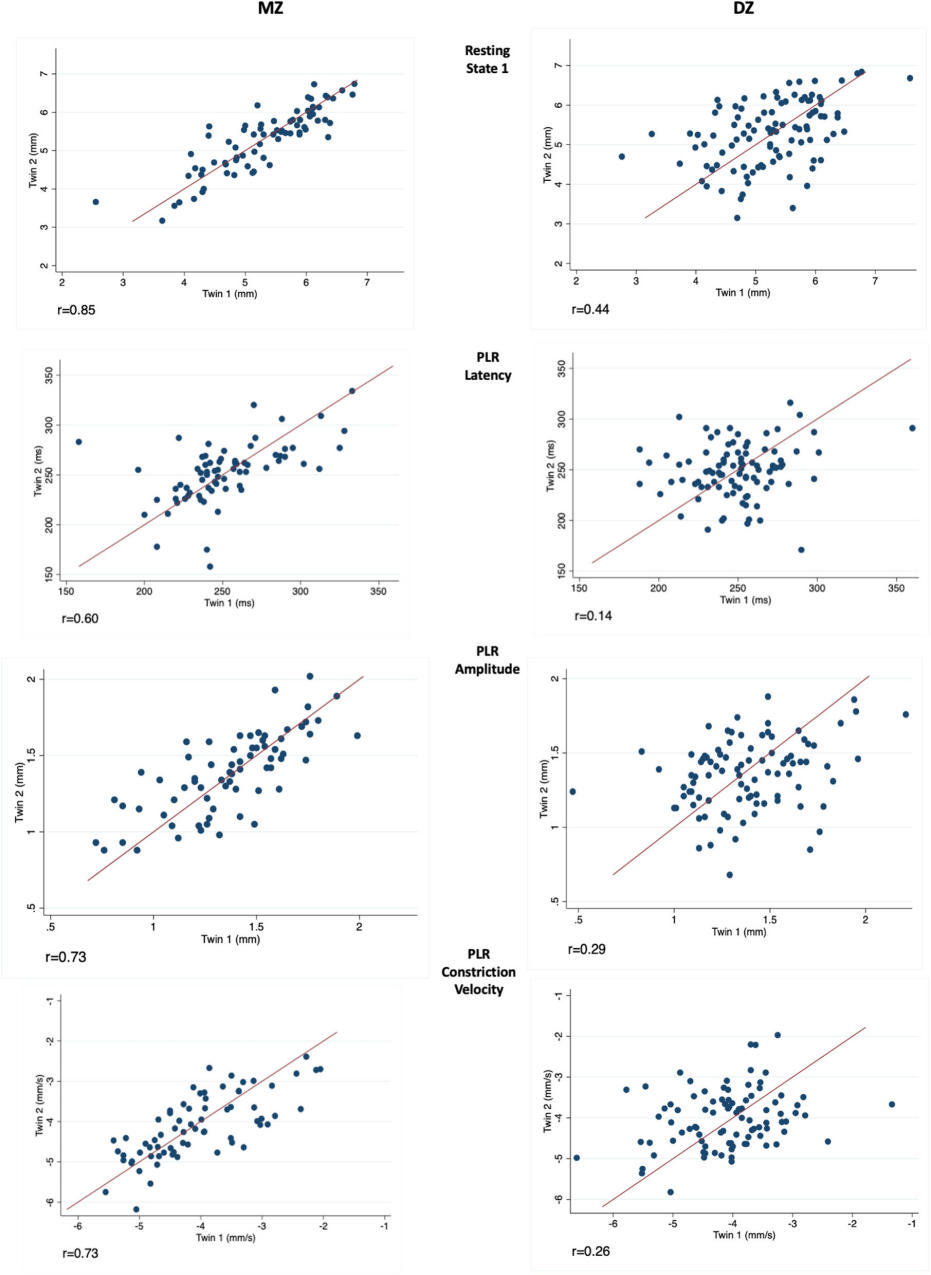
Measurements in twin pairs (twin 2 plotted against twin 1) for resting state 1 (Top), PLR latency, amplitude and constriction velocity (Bottom). Monozygotic (MZ) twin pairs are on the left and Dizygotic (DZ) on the right. Correlation coefficient (*r*) is given below for each parameter investigated.

Correlations between MZ-DZ pairs were calculated prior to the completion of twin modelling through calculating within-pair correlations by zygosity. Resting pupil size measurements demonstrated a stronger correlation within MZ twin pairs than DZ pairs at all four levels of illumination, and also at measurements of PLR latency and amplitude. The left eye resting state 1 (Dark) intra-pair correlation coefficient for MZ twins was 0.85, almost double that of DZ 0.44, suggesting a strong genetic effect on pupil size. Similar results were seen for dynamic parameters such as PLR amplitude, constriction velocity and pupil latency. Scatter plots depicting intra-pair correlation for MZ and DZ twins for the studied pupil parameters are shown in Figure 2.

### Estimation of Heritability

For the sample of complete twin pairs (*n* = 163) model fit statistics suggested the AE model provided the most parsimonious fit for all traits studied (Table 4). This would suggest the variance in resting pupil size, amplitude, constriction velocity and pupil latency are best explained by additive genetic and unique environment effects, whilst common family environment and dominant genetic effects could be dropped with no significant loss of fit.

**Table 4:** Results of twin model fitting for left eye only.

	**Model**	**Minus2LL**	**df**	**p**	**AIC**
Resting state 1	ACE	639.98	310	0.96	19.98
pupil diameter	ADE	640.02	310	0.96	20.02
(complete twin pairs = 157)	**AE**	**640.02**	**311**	**0.98**	**18.02**
	CE	672.15	311	<0.01	50.15
	E	747.73	312	<0.01	123.73
Amplitude	ACE	−16.64	286	0.22	−588.64
(complete twin pairs = 145)	ADE	−17.84	286	0.31	−589.84
	**AE**	**−16.64**	**287**	**0.31**	**−590.64**
	CE	−1.72	287	<0.01	−575.72
	E	29.41	288	<0.01	−546.59
Constriction velocity	ACE	608.92	286	0.17	36.92
(complete twin pairs = 145)	ADE	607.36	286	0.27	35.36
	**AE**	**608.92**	**287**	**0.24**	**34.92**
	CE	621.44	287	<0.01	47.44
	E	647.51	288	<0.01	71.51
Pupil latency	ACE	2,723.68	286	0.24	2,155.68
(complete twin pairs = 145)	ADE	2,722.27	286	0.36	2,150.27
	**AE**	**2,723.68**	**287**	**0.33**	**2,149.68**
	CE	3,300.08	287	<0.01	2,612.08
	E	2,739.84	288	<0.01	2,163.84

*The Akaike information criterion (AIC) is used to evaluate the most parsimonious model, which is highlighted in **bold**. For each model the minus 2 log-likelihood (minus2LL), and degrees of freedom (df) for model comparison are given. A, additive genetic effects; D, dominant genetic effects; C, common environmental effects; E, unique environmental effects (and measurement error)*.

Standardised parameter estimates for the best fitting models for the left eye can be seen in Table 5. This would suggest the left eye heritability of the resting pupil size in darkness (state 1) is 86% (95% CI 79–90%) with the remaining 14% (95% CI 10–21%) explained by unique environment. Similar results were seen for other resting states examined, with heritability estimated to be 76% (95% CI 65–83%) for resting state 2, 76% (95% CI 65–83%) for resting state 3 and 70% (95% CI 57–79%) for resting state 4, respectively.

**Table 5:** Standardised parameter estimates for the best fitting models for the left eye.

	**Left eye**			
**Measure**	**a^**2**^**	**95% CI**	**e^**2**^**	**95% CI**
Resting state 1 diameter	0.86	0.79–0.90	0.14	0.10–0.21
Amplitude	0.69	0.54–0.79	0.31	0.21–0.46
Constriction velocity	0.64	0.48–0.75	0.36	0.25–0.52
Pupil latency	0.40	0.21–0.56	0.60	0.44–0.79

Other dynamic variables investigated included PLR amplitude, constriction velocity and latency; the left eye heritability for these parameters was estimated to be between 69% (95% CI 54–79%), 64% (95% CI 48–75%), and 40% (95% CI 21–56%), respectively.

## Discussion

We have evaluated the relative contributions of genetic and environmental factors to resting pupil size under varying degrees of illuminance, and also pupil reactivity to transient light stimuli. Our results indicate that resting pupil size in complete darkness is strongly heritable with additive genes explaining up to 86% of the variance. As ambient light luminance was increased the heritability estimates fell, as expected given that many ophthalmic and external factors may affect pupillary constriction. A comparably lower heritability estimate was seen for dynamic characteristics such as PLR amplitude, velocity and latency, with variation due to additive genes ranging between 40 and 69%, respectively. This suggests that environmental factors are only responsible for 14% of the variability in resting pupil size within the population, and for between 31 and 60% of the variability in the PLR. It is noteworthy that baseline pupil diameter and reactivity have an equivalent heritability to many other highly heritable ocular traits such as central corneal thickness (95%) (28), optic disc parameters (66–73%) (29) and refractive error (77%) (30). We additionally examined for any association between pupil characteristics and iris colour, intra-ocular pressure and spherical equivalent. It has been reported that blue pupils may have a larger resting pupil diameter and be more dynamic (31, 32) however recent studies suggest this may not be the case (33, 34). Although our sample size was limited, we found no significant association between eye colours. We did however confirm a significant association with age for all characteristics (pupil resting diameter β = −0.02, *p* = 0.016), pupil light reaction amplitude (β = −0.01, *p* ≤ 0.001), constriction velocity (β = 0.03, *p* ≤ 0.001) and pupil latency (β = 0.98, *p* ≤ 0.001).

To date only two studies have attempted to evaluate the heritability of pupil size. The Guangzhou Twin Eye Study investigated the distribution and heritability of iris thickness and pupil diameter using anterior segment OCT (ASOCT). Their population of predominately East Asian individuals were aged between 8 and 16 years. Pupil diameter was measured between the most central points of the iris border. They estimated that genetic influences account for 63% of pupil size in a dark room (<5 lux) (35). This heritability estimate appears comparable to our findings. Our findings of high heritability are also comparable to that identified for pupil size after pharmacological mydriasis, which has been reported to be between 78 and 80% (intraclass correlation coefficients 0.82 in MZ twins vs. 0.39 in DZ twins) (11). To our knowledge no previous study has quantified the heritability of dynamic PLR measurements.

Although a number of factors that impact the pupil size and its dynamics have been well-described (36–39), there may be other influences including mechanical/anatomical factors. For example, iris thickness has been postulated as influencing the amplitude of pupillary constriction at high intensities using photic stimulation with blue and red light, possibly due to a passive resistance (39). Such local anatomy is highly heritable and would support the need for further genetic evaluation (35). Although limited, family-based genetic linkage studies have previously identified a congenital microcoria locus (40, 41), associated with maldevelopment of the dilator pupillae muscle of the iris and often associated with juvenile-onset glaucoma. Rare genetic mutations causing abnormal development of the anterior segment and iris are well-known, with a number of hereditary disorders being easily recognised through characteristic pupillary changes, including aniridia, iris colobomas, ectopic, scalloped or peninsula pupils, polycoria, congenital mydriasis, persistent pupillary membrane remnants and congenital miosis (42). The role of non-coding polymorphisms in these genes influencing normal pupil size variation and reactivity is unknown.

Additionally, given the potential environmental influence on PLR, a number of already established factors must be appreciated. In healthy individuals this may include: (a) retinal sensitivity to light (43); (b) any extrinsic factor, including those of dietary or pharmacological aetiology which may affect the function of retinal ganglion cells, in particular, mitochondrial function (44); (c) central influences modulating the excitability of neurones in the olivary pretectal nuclei and the Edinger-Westphal nuclei (45), (d) factors that may influence transmission across the neuro-effector junction in the iris sphincter muscle such as exposure to drugs or chemicals that modulate the availability and function of muscarinic receptors and (e) anything that affects the iris and its mobility, including the size of the lens, iris perfusion, oxygenation and any previous anterior segment pathology, anterior segment surgery or laser treatment (46).

Heritability is a population-specific factor and although our twin population data has been shown to be generalizable to the singleton population [as twins have similar morbidity and mortality to the rest of the population (47)], it consists predominantly of British women of European ancestry of a relatively narrow age range of whom are predominately in their sixties. Other limitations include that of volunteer or recruitment bias which could lead to an overestimation of the heritability of a trait. Additionally, the simultaneous effects of both shared-environment and gene/environment interactions cannot be considered concurrently without including additional siblings in the design.

The lower heritability of dynamic PLR characteristics in comparison to resting pupil size may be in part limited by measurement error, environmental factors or unknown shared family factors. Pupil latency, for instance, was seen to have relatively low heritability, however, we believe this is reflective of inaccuracy in its measurement. The pupilometer used possesses a more accurate spatial resolution in comparison to its frequency resolution. Potentially explaining any imprecise results. Given these limitations, we believe our results warrant further exploration on a larger scale, to determine if these conclusions are applicable to the wider population.

Future work should include genome-wide association studies to identify genetic polymorphisms associated with pupil diameter variance. A greater understanding of the genetic factors and biological mechanisms underlying pupil size and reactivity might allow greater clinical utility of assessing normal pupil size and reactivity in health and disease. Assessing pupillary responses can also be a useful clinical tool in monitoring certain neurological conditions or identifying those that warrant further investigation. As an example, unilateral macular diseases rarely show a relative afferent pupillary defect (RAPD) of more than 0.5 log units, thus if a measured RAPD is greater than this, additional pathologies such as compressive optic neuropathy must be considered (21). Additionally, given the widespread disparity of normative measures in the general population, the clinical significance of a solitary pupillary measurement may hold limitations, but this could be improved by obtaining serial measurements from the same individual over a period of time, therefore offering a surrogate marker of disease progression.

In conclusion, this study provides a current, well-phenotyped normative dataset of pupillary size and reactivity in a largely European female population. Twin modelling demonstrates that genetic effects are a key determinant of both resting pupil size, particularly in dim light, and reactivity characteristics including latency, constriction velocity and amplitude. The knowledge that pupil size and reactivity is predominantly genetically determined should direct future research into understanding functional mechanisms influencing pupil size and ultimately improve our ability to use these measures clinically.

## Data Availability Statement

The raw data supporting the conclusions of this article will be made available by the authors, without undue reservation.

## Ethics Statement

The studies involving human participants were reviewed and approved by King's College London - research ethics approval (EC04/015). The patients/participants provided their written informed consent to participate in this study.

## Author Contributions

FB and CH were involved in the conception and design of the study. FB and JV were involved in data collection. JV, CH, AA, FB, and KW carried out the statistical analysis and data interpretation, provided critical review of the manuscript, and contributed to the final write-up. AA and KW drafted the manuscript. All authors read and approved the final manuscript.

## Conflict of Interest

The authors declare that the research was conducted in the absence of any commercial or financial relationships that could be construed as a potential conflict of interest.
